# Pestilence and Plague

**Published:** 1879-11

**Authors:** 


**PESTILENCE AND PLAGUE** have destroyed millions of lives. In long ages past, they have half-depopulated cities, and decimated empires. Whence came they? From human dereliction, as all other curses and calamities come, and not from the Almighty's hand, because he promised Noah, "I will not again curse the ground any more for man's sake," and, as we paraphrase the assertion, "let him be ever so bad." Plague is from a Greek word meaning "to blow," as if it came on the wings of the wind. Pestilence is the Latin word which means the same thing. The "Great Plague" visited London in 1665, and the next year a terrible fire laid a very large portion of it in ashes, since when it has not re-appeared there. Erasmus wrote of England three hundred and fifty years ago, in the days of "King Hal:" "The floors of their dwellings are strewn with green rushes, which are allowed to increase, layer upon layer, for twenty years together, covering up bones, crumbs from the table, and other filth; and to this, and the general dirty and slovenly habits amongst the people, may be ascribed the frequent plagues in England." And this in Henry the Eighth's day! What a disgusting idea! Yet, in our own time, filthy cellars, dirty kitchens, and foul, dark closets, are the unsuspected sources of habitual sickness to whole households.

---

**We aim to show how disease may be avoided, and that it is best, when sickness comes, to take no medicine without consulting an educated physician.**

